# “These Girls Have a Chance to be the Future Generation of HIV Negative”: Experiences of Implementing a PrEP Programme for Adolescent Girls and Young Women in South Africa

**DOI:** 10.1007/s10461-022-03750-1

**Published:** 2022-07-06

**Authors:** Zoe Duby, Brittany Bunce, Chantal Fowler, Kim Jonas, Kate Bergh, Darshini Govindasamy, Colleen Wagner, Catherine Mathews

**Affiliations:** 1 Health Systems Research Unit, South African Medical Research Council, Francie van Zijl Drive, Parow Valley, Tygerberg, Cape Town, South Africa; 2 Division of Social and Behavioural Sciences in the School of Public Health and Family Medicine, University of Cape Town, Cape Town, South Africa; 3 Institute for Global Sustainable Development (IGSD), University of Sheffield, Sheffield, United Kingdom; 4 NACOSA (Networking HIV/AIDS Community of South Africa), Cape Town, South Africa

**Keywords:** Adolescent girls and Young Women, South Africa, PrEP, Implementation, HIV

## Abstract

Daily oral pre-exposure prophylaxis (PrEP) is highly efficacious for HIV prevention. Adolescent girls and young women (AGYW) have been prioritised for PrEP delivery in South Africa. A combination HIV prevention intervention providing integrated biomedical, behavioural and structural interventions for AGYW aged 15–24 in twelve districts in South Africa characterised by high HIV prevalence, was implemented 2019–2022. We conducted qualitative interviews to explore PrEP implementation experiences with 38 individuals involved in the implementation of the combination HIV prevention programme, including programme managers and project coordinators, health care providers / nurses, social workers, counsellors, peer group trainers and outreach workers. Narratives included various challenges associated with PrEP uptake, adherence and acceptability experienced by implementers. Barriers to PrEP acceptability included AGYW fears of side effects and preference for injectable versus daily oral PrEP; resistance towards PrEP from AGYW, communities and parents due to a lack of accurate information; PrEP stigma linked to associations with antiretrovirals and assumptions of promiscuity; and issues pertaining to parental consent. Additionally, implementers faced logistical challenges related to procurement, stockouts, and supply of PrEP. Findings highlighted the critical role of parental and community acceptability of PrEP for successful implementation. Overall, PrEP was perceived by implementers as a valuable HIV prevention tool. In order to ensure the accessibility of PrEP for AGYW in South Africa, efforts to reduce stigma and foster social support for PrEP use, campaigns to raise awareness, ensure positive framing of PrEP, and build community acceptability of PrEP, are needed.

## Introduction

South Africa’s human immunodeficiency virus (HIV) epidemic is both the largest in the world, and also the fastest growing [1, 2]. There exist significant differences in HIV incidence and prevalence by age and sex; women face disproportionate HIV burden throughout the life cycle, but this gender disparity is most pronounced among adolescent girls and young women (AGYW) aged 15–24 years, whose HIV prevalence is 3.3 times greater than their male peers (15.5% compared to 4.8%) [3–5]. South Africa’s epidemiological profile shows that while new HIV infections do occur in adolescent girls below 15 years, incidence is substantially higher in the 15–24 age group, given that the majority (92.4%) of AGYW have their sexual debut at 15 or older [4]. In light of this evidence, AGYW are regarded as a priority population in South Africa’s National Strategic Plan (NSP) for curbing HIV, TB and STIs: 2017–2022. There is an urgent need for effective female-initiated HIV prevention strategies targeted at AGYW, to reduce HIV incidence among this population [3].

Numerous sociocultural, behavioural, structural and physiological factors contribute to the disproportionate HIV risk that AGYW in South Africa face. Gendered power inequities are weighted in men’s favour, limiting AGYW’s power and agency to use condoms [6]. Additionally, gendered socio–economic power disparities contribute to AGYW’s vulnerability to HIV infection, through increasing their likelihood of engaging in transactional sex, condomless sex, and age-disparate sexual partnerships [7]. Girls and women often engage in sex that they do not want, do not like, or are not comfortable with, to maintain relationship security and to avoid violence from their sexual partners [8]. The lack of agency that AGYW have to control condom use, and the timing of sex, decreases their likelihood of success in using coitally-dependent HIV prevention products, indicating that other HIV prevention options, that confer AGYW with more self-efficacy and agency, are critical for this population [8, 9].

The benefits of pre-exposure prophylaxis (PrEP) for HIV prevention have been well-established [5, 10]. A daily dose of oral PrEP has been shown to be highly efficacious, giving > 90% protection against HIV acquisition when taken with high adherence, and has thus been hailed as one of the most important biomedical strategies in the toolkit for HIV prevention [10–12]. In light of this, the South African Health Products Regulatory Authority (SAHPRA) approved the use of daily oral PrEP in 2015, for adults and adolescents > 35 kg [5, 13]. Specifically, evidence shows that PrEP offers powerful protection for women, and therefore AGYW have been prioritised for PrEP delivery [13, 14]. PrEP effectiveness is dependent on access, adherence, and persistence [10]. Importantly, access, use, and adherence to PrEP also requires substantial commitment and support from health care providers [15].

Previous studies have examined issues relating to acceptability of PrEP and issues related to adherence amongst AGYW in South Africa [13, 16], but few studies have examined the perspectives and experiences of people engaged in the implementation of PrEP programmes. Data relating to the experiences of implementing PrEP programmes for AGYW are limited, particularly on approaches to effectively reach and deliver PrEP to AGYW in high HIV burden communities [10]. Considering lessons learnt from PrEP demonstration projects for South African AGYW is critical to inform future PrEP roll-out to this population [14]. In the analysis presented in this paper, we sought to explore qualitative narratives comprising the experiences of implementing a PrEP programme for AGYW in South Africa, describing successes, challenges, and lessons learnt. Included in our investigation was an assessment of the acceptability of the PrEP programme. Acceptability has been defined as the perception among intervention beneficiaries and implementation stakeholders that a given intervention and its activities are agreeable or satisfactory [17]. Acceptability is not simply an attribute of an intervention but is rather a subjective evaluation made by individuals who experience or deliver an intervention [18]. Acceptability is an important issue to consider in the development, evaluation and implementation phases of interventions, and should be assessed based on stakeholders’ knowledge of, or direct experience with various dimensions of the intervention [17, 18].

## Methods

### Background to the intervention

Due to emerging epidemiological data reflecting the enormous burden of disease on AGYW, the Global Fund to Fight AIDS, TB and Malaria (Global Fund) provided funding for a new programme that focused HIV prevention on AGYW. The AGYW Programme (2019–2022) offered an age-tailored combination intervention for AGYW aged 15–24 in twelve districts in South Africa characterised by high HIV prevalence. Combination HIV prevention interventions, merge biomedical, behavioural and structural interventions for combined delivery, and have been identified as one of the key strategies for reaching the 90-90-90 targets and achieving the Sustainable Development Goal (SDG) of ending the HIV epidemic by 2030 [19]. In line with South Africa’s National Strategic Plan for HIV, AIDS and TB, 2017–2022, which prioritises AGYW age 15–24 for PrEP, and coinciding with the National Department of Health (NDoH) scale up and roll out plans for AGYW using National Guidelines, the AGYW programme intervention included PrEP demand creation, initiation onto and monthly provision of PrEP based on a risk assessment, or referral to the closest health facilities if PrEP was not provided by the sub-recipient implementing organisation (SR). PrEP provision occurred through selected NDoH facilities and community-based satellite sites. Clinic and outreach services were intended to be equipped to initiate PrEP for AGYW beneficiaries who tested HIV negative and were assessed to be ‘at risk’ at these sites. Guided by the South African Children’s Act 38 of 2005, which allowed mature adolescent girls to self-consent to PrEP as early as 12 years of age [20], AGYW were able to initiate onto PrEP without parental consent. Early on in the grant implementation period, each principal grant recipient (PR) developed provincial PrEP Implementation plans with NDoH, to ensure harmonisation of Global Fund PrEP targets, roll-out and delivery methodologies with Departmental procurement, strategies and protocols. Representatives from the intervention team also sat on South Africa’s national PrEP Technical Working group throughout the grant period.

### Study procedures

The HERStory2 study was a process evaluation of the AGYW combination intervention. The sample for the evaluation study was drawn from 6 of the 12 sub-districts in which the intervention was being implemented, comprising two sub-districts per PR, as follows: Klipfontein, Cape Town (Western Cape), King Cetshwayo (KwaZulu Natal), Ehlanzeni (Mpumalanga), Rustenburg (North West), Nelson Mandela Bay (Eastern Cape), and Thabo Mofutsanyana/Dihlabeng (Free State). Individual in-depth interviews (IDIs) were conducted in the period from November 2020 and March 2021, with 38 individuals involved in implementing the intervention in various capacities. We purposively sampled a range of implementers, including programme managers and project coordinators, health care providers / nurses, social workers, counsellors, peer group trainers and outreach workers^1^. Semi-structured interview topic guides were used to explore barriers and facilitators for implementation. Emphasis was placed on all aspects of intervention delivery, feasibility and acceptance. We also examined contextual issues that may have shaped the delivery of the intervention. Interviews were conducted telephonically, in participants’ language of choice, by trained interviewers fluent in several of South Africa’s official languages. Interviews were recorded, transcribed verbatim or translated into English as necessary, and reviewed for accuracy.

Following a cyclical process, a team of three analysts engaged in immersive iterative thematic analysis of the data. A set of pre-determined deductive code types based on the topics included in the interview guides, were built upon through the development, refinement and expansion of codes. Emergent key themes were identified in initial transcript readings, and evolved iteratively through a deductive and inductive process with subsequent readings. As concepts and themes emerged, the team collaboratively reviewed and discussed them, returning to the raw data, and refining themes through team consensus. Analytic memo-ing was conducted in parallel, capturing analysts’ reflections, interpretations, and developing insights. Memo-ing allowed enhanced data exploration, enabling analysts to articulate and communicate interpretations, connect to wider theory, summarise emergent findings [21]. Further details on the study methods can be found: https://www.samrc.ac.za/intramural-research-units/HealthSystems-HERStory.

#### Ethical considerations

The South African Medical Research Council Research Ethics Committee (EC036-9/2020) approved the study protocol. All participants provided informed consent for participation.

## Findings

### Implementer experiences with PrEP demand creation and acceptability of PrEP amongst AGYW

Successful implementation of any PrEP programme relies on demand creation. Implementers’ narratives on AGYW acceptability of PrEP were varied, ranging from enthusiasm, to scepticism and hesitancy.

*To be honest, they (AGYW) are a bit sceptical about PrEP… I don’t know if it is caused by the fact that it is still something new, or they don’t want it, or they need more information… but they are really sceptical… they (AGYW) need more information before they jump in. (School-based Implementer)*

Reasons for AGYW resistance and scepticism towards PrEP posited by respondents included prevalent misinformation about PrEP amongst AGYW and in the broader community, PrEP stigma, a lack of understanding about the difference between PrEP as prevention and antiretrovirals for HIV treatment (ARVs), unsupportive parents, and fear about negative side-effects.

*AGYW would say they were told that PrEP is like ARVs, which is due to lack of information about PrEP. Others would say they want to first ask permission from their mothers… I once had a case, this girl was willing to take PrEP, but it was not good for her (she experienced side effects)… so she stopped taking it… she told me that her entire body got swollen. (Clinic-based Implementer)*

Respondents described prevalent misconceptions around PrEP in the community, relating to the lack of awareness that while PrEP is an antiretroviral and can be taken as treatment by HIV positive people, it can also be used as a prophylaxis by HIV negative people. The quotation below illustrates how these misconceptions generate anticipated stigma among AGYW, thereby serving as a barrier to PrEP use.

*One thing that needs to be improved… they (AGYW) are not getting in-depth education about PrEP… there’s a lot of gaps… things they don’t understand – that it’s ARVs… the drug that they’re using… we need to iron out and explain: “this is an ARV”… because some of them, adolescents, they take this ARV, they go home and the mother knows, “oh, this is ARV”… and now it becomes a problem. “Why are you taking HIV medications?”… they end up getting stigmatized and being accused of having HIV… so that makes them (AGYW) very scared “my parents will think I’ve got HIV”. (Health care provider)*

Some respondents also suggested that since many AGYW in these communities live in circumstances of poverty, competing demands within AGYW personal life contexts mean that other basic needs take priority over HIV prevention.

*When you come in offering HIV testing and PrEP… for them it’s like “I’ve got bigger things to worry about than this PrEP of yours”. (Health care provider)*

Implementers described resistance to enrolling on the PrEP programme from AGYW who feared negative side-effects of PrEP, due to rumours and stories circulating.

*People do talk among themselves. Some would say that they get a rash, some would say that they can’t sleep… they will go around saying “these pills!”… So, most of them (AGYW) will say, “no I don’t want it, because this is what happened to my friend, so I don’t want it”. (Outreach/Mobile service provider)*

Even amongst those AGYW who were not experiencing side effects from PrEP, adherence was described as a key challenge. Implementers expressed frustration over these challenges, acknowledging that AGYW need to be motivated to adhere.

*Retention in the PrEP component (is a key challenge)… Yes, we are getting people inside the programme, they are taking PrEP, but they are not returning… if you initiated ten girls in a certain area… Your retention should be ten, because as long as you have reckless behaviours (girls need to be protected)… girls should be saying to themselves “OK I am having unsafe sex, but at the same time, yes I do want to live a longer life, a productive life… Then I will be taking my pre-exposure medication”… but that is not happening… What are we doing wrong? …is it the programme that is inconveniencing the girls? …is it the medication? Are there side effects? … Some of the challenges are difficult, things that we cannot do anything about, because if a person doesn’t have the will… there’s nothing much we can do. (Health care provider)*

One explanation for poor adherence to PrEP that implementers provided, was that AGYW find it hard to stick to the daily tablet dosing regimen.

*Most of them (AGYW) have said, “no I don’t have a problem with the medication… It’s just that I really don’t like taking medication every day”. (Health care provider)*

Implementers expressed the view that since AGYW are accustomed to and familiar with contraceptive injections already, AGYW would be more willing to have an injectable HIV prevention product rather than take daily oral tablets.

*The PrEP injection… caused a buzz (excitement)… girls were asking us about the injection and we told them “no, the injection is still under investigation”… (AGYW) are willing to take injection for 3 months… because they are already coming for family planning injection… so it will be convenient to get an injection every 3 months… They say taking medication every day is not on. (Health care provider)*

Having to take a daily prevention pill was viewed by AGYW as onerous as having to take daily ARV medication; implementers felt that offering PrEP as an injectable would address these barriers to PrEP interest amongst AGYW.

*They (AGYW) don’t want PrEP, they would have preferred PrEP to be an injection because they feel like they are eating treatment (ARVs) every day and yet they are not HIV positive, even though they need it (to be protected)… they wish it was an injection as opposed to taking pills every day. (Social worker)*

Implementers attempted various strategies for improving acceptability and uptake of PrEP amongst AGYW, including PrEP awareness events and campaigns, and the use of ‘PrEP ambassadors’ – AGYW peers already initiated on PrEP – who would provide encouragement to other AGYW.

*We were struggling to get girls to be on PrEP, so what we have decided to do is to use their own peers that are on PrEP already, to say “you are PrEP ambassadors, go and recruit your own peers to be initiated on PrEP”. We’ve seen that the turn-up of girls who are interested in PrEP is amazing because of their peers… it’s a very nice opportunity for them… those girls become ambassadors of PrEP to say (to their peers) “there’s a benefit, you take PrEP, you remain on PrEP, you protect yourself from being infected with HIV”. (Programme Manager)*

Uptake of PrEP was dependent on community level factors, and on relationships. Good working relationships and collaboration between community-based implementers and NDoH clinic staff could promote uptake of PrEP, with the community-based nurses referring AGYW to the NDoH facilities offering PrEP for monthly provision.

*The minute you tell them about PrEP they are keen to take it and keen to protect themselves more… Even in the clinic, they tell us, “we have got four clients for you”. (Social auxiliary worker)*

However even when AGYW were successfully enrolled into the programme and initiated on PrEP, adherence was a major challenge, with AGYW discontinuing or not taking PrEP as prescribed. Implementer respondents listed various reasons for AGYW discontinuing PrEP including short supply at clinics, unsupportive parents and communities, COVID-19 regulations limiting access, experiencing negative side-effects or hearing about bad experiences from peers. One view expressed by implementer respondents was that some of AGYW enrolled into the PrEP programme may have been attracted by incentives, for example caps (hats), received once initiated on PrEP, but had no intention of continuing their PrEP usage.

*PrEP is very challenging because even when you explained about PrEP… how it is taken… they (AGYW) do not take it… The number of initiations and the number of follow ups is not the same… Young people are just taking it for fun… they just show up and say “I heard there are pills”… We were giving them caps (hats) after taking PrEP. So one would take PrEP because they want a cap… (but) they don’t come back for a follow-up, they just take the PrEP and never use it. (Health care provider)*

### Community acceptability of PrEP

In implementers’ experiences, community views on PrEP provision to AGYW were mixed. Respondents highlighted the challenges that they had faced in gaining community acceptability. In some cases, implementers working in schools faced resistance towards PrEP from educators, creating a restrictive environment for educating AGYW about PrEP in the school setting.

*Since I’m working in a school… there are things that you cannot talk about inside the school… so I have never had a situation where I can tell a girl about PrEP. (School-based Implementer)*

According to implementers, disapproval of PrEP in the community has foundations in the belief that it encourages AGYW to take risks, be sexually active and promiscuous.

*People were saying that PrEP is condoning the kids to live recklessly. (Social auxiliary worker)*

It was also noted that in communities with a strong religious presence, there tended to be resistance to PrEP from faith leaders, who asserted that promoting PrEP was counter to their religious values, promoting and encouraging sexual activity amongst young people.

*The community were very negative saying that we are telling the young women to have sex. They were saying “you are telling them that you must wear a condom and that there is this pill that prevents HIV, so you are basically telling our children to go and have sex”. (Outreach worker)*

According to implementer respondents, in some communities the views of religious leaders had the potential to derail the PrEP programme.

*Such people preach and preach… people spend most of their time in their churches, so they have a big effect. When you offer PrEP to a young girl, they go around saying at the church “no, no, no those things are bad… ARVs, you must not take them”… there are all of these myths around HIV, so these people are making it difficult for us to convince our young girls who are really sexually active and not taking care of themselves, to have a little bit of protection… they are making it really hard for us to reach our young girls. (Outreach worker)*

However, there were also cases reported where church leaders were supportive of the programme and were allowing implementers to operate out of church facilities.

*Most of the people in the community are willing to work with us… sometimes we use the church building… so they are really being hands-on, because they understand what the program is about… we receive positive energy from them… the managers do get assistance from the church leaders. (Monitoring and evaluation staff)*

Implementer respondents emphasised that in general, community members are misinformed about PrEP. They cited several myths relating to PrEP, including that it causes infertility, results in termination of pregnancy or in HIV infection. A few cases in which people contracted HIV whilst being on PrEP, had also negatively affected community acceptability of PrEP. Information regarding whether these people were taking PrEP consistently and correctly was often not discussed alongside these unfortunate cases, when community members speak among themselves.

*People that are in the townships… you find that they have the wrong information… about PrEP, they will be saying, “oh if you are eating PrEP you end up getting HIV”… now you have to explain why some people use PrEP but they end up getting HIV positive. (Outreach worker)*

A common explanation for the lack of community acceptability of PrEP provided by implementers was the failure to differentiate between PrEP for prevention of HIV infection for HIV negative people, and antiretrovirals for the treatment of people who are HIV positive.

*They think PrEP is the same like ART (antiretroviral therapy). They think that we are giving the girls ART while they are still negative, because they don’t get much information about what PrEP is. They just take the information from the kids or whatever… They have that negative view of PrEP. (Health care provider)*

Misperceptions and a lack of understanding about PrEP in communities was believed to be fuelled by accessing inaccurate information online and on social media.

*They were saying that “PrEP is ARVs… why are you giving ARVs?”… And they will not give you the chance to explain what is PrEP… I just tell them that they mustn’t believe everything that is being posted online. (Counsellor)*

### Parental acceptability of PrEP

Implementers listed various factors negatively impacting on parental acceptability of PrEP. As AGYW were able to enrol on PrEP without parental consent, parents felt poorly consulted. A combination of being poorly consulted and a lack of information about PrEP, resulted in situations where parents would discover their daughters’ PrEP tablets, and accuse them of being HIV positive without having disclosed their status.

*You would initiate girls on PrEP, and then when they go home, the parents would be like, “what drugs are these? You are lying, you’re HIV infected”. (Programme manager)*

These dynamics created family conflict and anxiety amongst AGYW, leading to PrEP discontinuation.

*The children’s parents… some of the reasons that we get for stopping PrEP… when a parent sees that tin (tablet container) they become alerted that “my child is HIV positive… what is going on?” …there are misconceptions or misinformation from the parents. So, when a child says “no mom, I am taking this pill to protect myself from getting HIV”, parents are still using knowledge from the olden days… during the dark ages of HIV. (Health care provider)*

Some of the parents of AGYW who were HIV positive and taking ARVs themselves, were hesitant about having their daughters initiate on PrEP, given the misinformation about it.

*They are not getting in-depth education about PrEP, that’s the only problem… there’s a lot of gaps… things that they don’t understand. They think that it’s ARVs, actually the drug that they (HIV positive parents) are using themselves, so that is something that we need to iron out and explain… some AGYW take this ARV (PrEP), they go home and the mother knows, “oh, this is ARV”… and now it’s becomes a problem. “Why are you taking HIV medications?” (Health care provider)*.

In addition for PrEP being mistaken for HIV treatment, respondents described situations in which parents would mistake PrEP tablets for an abortion pill, which also created conflict.

*Parents were saying that we give their children PrEP so that they will abort… (it’s a) lack of information. They did not know the purpose of PrEP… if we told the parents what PrEP is, then we would be able to sell the idea to the children… Parents had… only heard rumours about PrEP and they assumed that PrEP is meant for abortion… they assumed we teach their children about sex and how to do abortion. (Social auxiliary worker)*

Illustrating the level of influence that parents have over their children’s’ decisions and behaviours, respondents described scenarios in which AGYW who had been initiated onto PrEP would discontinue due to parental pressure and a lack of understanding on the parents’ part about PrEP.

*Some of the kids that stop taking PrEP, it’s because of the information they got from their parents… and a parent’s voice is the most powerful over any other person, it’s the final word. So how do you change mother’s mentality, if the mother was not informed. (Health care provider)*

In addition to a lack of knowledge and understanding about PrEP, another common theme that emerged in explanation for the lack of acceptance of PrEP among parents related to the issue of parental consent. Implementers described scenarios in which AGYW expressed willingness to take PrEP but were reluctant to discuss with parents. The issue of parental consent around AGYW PrEP use presented an ethical and legal dilemma for implementers.

*Some parents say “how can you give my child ARVs without even my consent?”… those are like those ethical dilemmas where now, it’s culture versus the medical field… some of them (AGYW)… are 15 (years old)… and the mother will come and fight you: “Why are you giving my child (medications) without my consent?” (Health care provider)*.

Some respondents felt that parental resistance was caused by a generational gap, and the perception that the intervention was promoting sexual activity.

*Some of the parents are old and they don’t understand… They do not know what PrEP is. They see a child taking pills and they don’t understand… Imagine if you’re sixty years of age and your child is taking pills you know nothing of and you don’t understand… So the parents refuse their children to be involved… they say were are teaching their children so be sexually active… that is why we are giving them PrEP. (Social worker)*

### The need to address barriers towards PrEP acceptability

The general perception amongst implementer respondents was that there was a need for concerted efforts to raise awareness around PrEP, targeting parents and community gatekeepers especially, to create a conducive and supportive environment for AGYW to disclose their PrEP use, and facilitate adherence.

*There is a need to go out and explain what PrEP is actually, what the drug does. Because people know it’s the PrEP, but they don’t know it’s an ARV, so that makes the girls very scared like “…my parents will think I’ve got HIV”…the girls end up coming… and collecting their medication and having to change the container and say “I’m taking vitamin C or supplement of some sort” instead of talking freely with their parent. (Health care worker)*

Implementers also felt that parental support for PrEP use amongst AGYW would contribute to efforts to improve uptake and adherence amongst AGYW.

*Mothers would want to push (encourage) their children to be in our project, because we talk about prevention… I used to explain to them that if as a mother, you are not open to your child… then it’s going to be difficult… it becomes much easier if the mother would be asking the child if she had gone to the clinic, “did you take your PrEP?”…to remind her. (Peer group trainer)*

Some of the implementers felt that resistance to PrEP was caused by failure on the part of programme implementers to properly educate AGYW, their parents and the community more broadly about PrEP. Overall, implementers felt engagement with parents was insufficient, contributing to parents being poorly informed about PrEP. In some cases it was felt that AGYW were tasked with sharing information about PrEP and the programme with their parents, which many AGYW were ill-equipped to do successfully.

*We work with our advocacy partners to say, this is an issue, can you please go back to the community and educate them about PrEP, so that we close the gap, because there’s a gap between the parents and the girls… the girls know what PrEP is, however the parents don’t. (Programme manager)*

Implementers felt that the acceptability and uptake of PrEP could be improved through increased efforts to engage parents in PrEP promotion and education.

*Some kids they are keen to take PrEP but the parents don’t know anything about it. So, it is better to involve the parents… let them understand. (Social auxiliary worker)*

### Successful strategies for increasing PrEP acceptability

Some implementers described strategies they had in place to try and engage parents and community members to dispel beliefs that they were promoting AGYW sexual activity, and rather that they were promoting self-care and health empowerment.

*We are going to have a dialogue with AGYW. We are going to invite parents and stakeholders so that parents should see what information is given to their children when we talk to them… there are some misconceptions in the community that we teach their children to have sex and such things… parents do not understand what we mean when we talk about PrEP and such stuff… they think that we encourage their children to have sex… (dialogue) will help a lot so that they could see that we encourage the kids to lead a healthy life and take care of themselves, most importantly on their sexuality. (Social auxiliary worker)*

Through engaging with parents, providing them with information, implementers were successfully able to overcome some parents’ initial resistance to their daughters being on PrEP, and gain their acceptance.

*The challenge was because PrEP is an ARV medication, and some of the parents had that medication at their home. So, they thought that we are giving their kids ARVs. We had to do a lot of explanation to make them understand how these things work… Some parents still don’t allow us… But through more engagement, parents are more understanding and some parents are even calling us to initiate PrEP for their kids. (Health care provider)*

In some communities, there seemed to be a sense of acceptance of the PrEP programme among community members and parents of AGYW, in recognition of the HIV risks AGYW face.

*They (community) like this thing about PrEP. They know that a lot of young people sleep around without using a condom. (Outreach worker)*

An appreciation of the protective properties of PrEP was a key factor in parental and community acceptance, with AGYW safety and health being a key priority.

*They are very supportive… the parents are the ones who bring the young women to us. They are excited about PrEP because they know that it will keep the girls safe even if they engage in sexual activities. (Project coordinator)*

There were even cases reported in which parents had come into the programme offices to share their positive views on PrEP.

*There was one parent… who came in our office and told us that the child changed drastically… the child is now educating the mother about PrEP, about preventing yourself, taking care of your health and about everything… the mother was really, really happy. (Social auxiliary worker)*

One suggestion to improve AGYW ability to take PrEP was through working to build relationships between AGYW and their parents to facilitate open communication and foster support.

*When you are working with a young girl, the best thing that you should do is you must involve the parents… There are some parents that feel like when you give their children PrEP you are promoting them to have sex and to live recklessly. But if we can make them aware and sensitise them about what it is that we do and the advantage of the program and letting their kids to be in the program… let the girls build a relationship with their parents… like maybe a group support where the mother and the child become friends. If they can get to that level that they talk about everything it becomes easier for us to work with them because some kids they are keen to take PrEP but the parents don’t know anything about it. So, it is better to involve the parents… remember that it is not easy to change someone’s mindset about certain things, so you need to take them step by step so they can understand better. (Social auxiliary worker)*

Successful strategies for engaging parents and getting their buy-in included building connections through school based parents’ meetings.

## Acceptability of the PrEP programme among implementers

Most of the implementers were very positive about the PrEP programme and its potential impact, expressing their excitement at being involved in the roll-out of this important new biomedical prevention product.

*Our main programme entails offering PrEP to HIV negative girls… there was excitement for the fact that now these girls have a chance of being the future generation of HIV negative… doing something about it and bringing the services to their own level… that was the best part of it… Even though we are working in difficult conditions… it was really kind of exciting. (Health care provider)*

Respondents emphasised how critical PrEP was in protecting AGYW from HIV infection, even in the absence of behavioural and structural changes.

*PrEP is assisting us because if a young woman has initiated PrEP… HIV will decrease, even though she can have blessers (engage in transactional sex/relationships), but she will not be infected with HIV. So, we are getting there, what will follow will be an HIV free generation. (Peer group trainer)*

Implementers believed in the importance of increasing AGYW access to PrEP, to reduce HIV incidence in this vulnerable at-risk population.

*My wish is that all of them can be a part of PrEP, because it is a part of the prevention of HIV… Especially now that the topics now are about rape and killing of AGYW most of the time. So, if they can be part of PrEP, we will know that we can be able to fight. (Counsellor)*

Since implementers believed that PrEP provided an opportunity for an HIV free generation, they expressed frustration over the lack of interest in taking PrEP amongst AGYW, viewing it as a wasted opportunity.

*What is disappointing is the fact that retention for PrEP is not as I thought, because you know when there is a new vaccine for saving lives, you have your own perceptions, especially with a South African background of HIV. We have family members that have passed on… we have families brought up by children (child-headed households). So you assume… when this medication comes in everybody… the youth of South Africa, will be aware of the fact that it’s their chance… “This is our opportunity… (thinking of) our parents or the previous generation who died of HIV, at least we are going to be safe”… But then in practice, it’s totally different… in South Africa we have a generation of people… it’s not as if people are not aware of the information out there… They are aware but it’s a matter of choosing whether you want to live a better life of HIV free or you choose to be HIV positive… at the same time you can’t force people to do something if they are not ready. You can’t coerce them. (Health care provider)*

In the interviews, several implementers noted that the rollout of the PrEP programme was slow, and that there were various logistical challenges regarding ensuring an adequate and consistent supply of PrEP. There were challenges in working with external service providers to procure and distribute the pharmaceutical supplies to the facilities where PrEP was to be provided, namely selected government health facilities and community-based satellite sites. Although costs of PrEP were covered by funders, sourcing the medication supplies via the district levels of the NDoH was described as a complex process. The limited availability of PrEP affected the participation of some AGYW, who had initially agreed to participate in the PrEP programme, but when they could not be initiated onto PrEP due to stock-outs, subsequently lost interest.

*We had challenges with PrEP, because it was in short supply at the clinics. It was allocated in one facility, but you will find that in that facility it is finished. You know how government works, they take time to order… So these young women have already been recruited and given an appointment date, and when they come and find that we don’t have, you give them another appointment date, they will no longer be interested, so that was the challenge. But now the Department of Health has opened more facilities that distributes PrEP, things are now flowing, and we are working nicely. (Project coordinator)*

Implementers also explained that insufficient and inconsistent supplies of PrEP at clinics meant that AGYW were deterred from initiating onto PrEP, or some of those who had been initiated later discontinued PrEP.

*We offer these young girls PrEP… but right now we honestly don’t have any… so it becomes hard for me to do my job because I am promising these young girls, “we have this that can prevent HIV”… We gave them some but the next time they come to collect, we don’t have any, and then it comes to the second month and we still don’t have any… it makes my job difficult… it makes me feel like, I don’t want to go out there now and say things that make it look like I am lying. (Outreach worker)*

Overall, restrictions and processes of procuring and distributing PrEP were described as challenging, characterised by stockouts, insufficient supplies, or barriers within NDoH facilities.

## Discussion

Implementer narratives relating to experiences and perspectives of the PrEP programme for AGYW provide valuable insight into the lived experiences, challenges, successes and lessons learnt of implementing such an intervention in the South African context. Several challenges associated with PrEP uptake, adherence and acceptability were described, including AGYW fears of side effects and preference for injectable versus daily oral PrEP. Challenges relating to AGYW, community and parental resistance to PrEP described by implementers were due to a lack of accurate information about PrEP, and PrEP stigma linked to associations with antiretrovirals and assumptions of promiscuity. Linked to the importance of parental buy-in for PrEP was also the issue of parental consent for enrolling AGYW into the PrEP programme, described by implementer respondents as an ethical and legal dilemma. Findings highlighted the critical role of parental and community acceptability of PrEP as an enabler to successful implementation. Findings indicate that collaboration and good working relationships between implementing organisations and public facility clinic staff can promote and enable PrEP programmes, enabling successful PrEP demand creation, provision and retention. However, even where relationships were good, there were issues with stock-outs at the facility level, which threatened to undermine the PrEP programme. Overall, implementer respondents believed that PrEP is an important and valuable tool in HIV prevention, and were excited about being part of an innovative programme.

Implementer respondents reported considerable challenges related to PrEP retention, noting that although a high number of AGYW were initiated onto PrEP, few were successfully retained​. Published findings from PrEP programmes for AGYW implemented in African countries have demonstrated continuation of PrEP as a key challenge, showing substantial drop-off in the first few months, with the majority discontinuing within the first six months [22]. Contributing factors could include changing risk perception and motivation due to changing sexual relationships and perceived prevention needs, lack of familiarity with antiretrovirals for prevention, lack of social support, PrEP stigma, pervasive concerns about side effects with PrEP, and perceptions that required clinical engagement is overly burdensome [11, 22, 23], many of which were described by respondents in our study.

As with any biomedical intervention, logistics of supply chain management, including procurement and distribution, can be challenging. Implementers described how the roll-out of the PrEP programme faced various delays due to challenges in supply of PrEP through the National Department of Health, which negatively affected AGYW participation. A key challenge in community-based PrEP delivery relates to commodity supply, not only of PrEP, but also of HIV testing kits and laboratory supplies. Provincial departments of health in South Africa often experience stockouts and insufficient supplies. Medicine stockouts remain one of the key constraints in the delivery of effective healthcare across South Africa; a study in 2014 showed that a quarter of facilities, notably unevenly distributed across provinces and districts, experienced stock outs of ARVs [24]. Challenges with ensuring consistent supply of medications through community clinics have been well documented in the South African context, with a history of linkage/continuity challenges in the delivery of HIV and TB treatment from static clinic sites [10]. Our findings add weight to the evidence base showing that the successful implementation of PrEP programmes at a national level requires collaboration and on-going engagement with communities, the government health sector, and non-governmental organisations, inclusive of the promotion of PrEP outside of the clinical setting as a means of increasing acceptability and achieving successful uptake and scale-up of PrEP services [9].

Part of successful demand creation involves making PrEP as attractive as possible to AGYW. Implementer respondents in our study described AGYW enthusiasm over the idea of injectable PrEP, suggesting that given their familiarity with injectable contraceptives, AGYW would be more willing to have an injectable HIV prevention product rather than take daily oral tablets, and that this might help to circumvent some of the issues relating to adherence and PrEP stigma. AGYW preference for a longer-lasting injectable PrEP over a daily oral dosing has been reported in other South African studies [1, 13].

In addition, implementer respondents in our study spoke about the use of incentives as motivations for AGYW to join the PrEP programme, but described scenarios in which AGYW would join the programme in order to get the incentive, but without any intention of staying in the programme or adhering to PrEP. It is important to consider strategies to ensure continuation of care amongst AGYW. As our data shows, material incentives such as clothing are useful for attracting AGYW to the programme, but there may be more need for rewarding longer term persistent adherence in ways that are responsive to context and to the needs of AGYW. Previous studies examining AGYW motivations and adherence have reported that receiving incentives did not significantly impact AGYW motivation to use PrEP, and that the desire to take PrEP in order to be a ‘responsible young woman’ was more of a motivating factor [11, 13]. As evident in our findings, factors such as food insecurity and ability to meet basic needs may affect AGYW motivation to engage in HIV preventative behaviours; those AGYW who are experiencing economic and livelihood challenges that are deemed more urgent, may be less inclined to take PrEP. Given that there will be diversity amongst AGYW in terms of motivations to use PrEP, it would be important to tailor adherence support approaches, including appropriate nudges and incentives, to cover a broad spectrum [11, 13].

Emerging clearly in the data, was the critical role of parental and community acceptability of PrEP as an enabler to successful implementation. Where parents of AGYW had been insufficiently engaged or consulted, programme implementers experienced hostility, especially where AGYW had been initiated on PrEP without parental consent. Where there was parental acceptability for PrEP usage among AGYW, implementation was more likely to be successful, and AGYW more likely to be retained into the programme, as parents/caregivers encouraged AGYW to keep attending services and advocated for the programme among other community stakeholders. Benefits of having parental buy-in for PrEP were evident in instances described by implementer respondents in which parents recognised the protection PrEP provides and encouraged their daughters to enrol in the programme. The issue of parental consent for enrolling AGYW into the PrEP programme was described by respondents as an ethical and legal dilemma. Family tensions and conflict arose when parents discovered their adolescent was taking PrEP without their knowledge, creating challenges even when legally, consent from parents is not required. This point is discussed in the literature on the ethical and legal issues of requiring parental/guardian consent for PrEP amongst adolescents, which can inhibit access and uptake for the reason that requesting consent entails disclosure of sexual activity [25, 26]. Debates on parental consent for PrEP centre around interpretation of the South African Children’s Act 38 of 2005, which makes provision for children from the age of 12 to give their own consent for medical treatment; in this case, if PrEP is interpreted as ‘medical treatment’, then self-consent for PrEP is permissible for persons over 12 years, if they have the mental capacity and maturity to understand the benefits, risks, social and other implications of the proposed treatment [5, 20, 27]. Opponents to this view posit that whilst the Children’s Act provides clarity on consent to most medical interventions for children under 18 years, it does not directly address the age at which adolescents might self-consent to non-specified preventive interventions such as PrEP [5]. However, PrEP proponents argue that although PrEP is not expressly referred to in the Children’s Act, it should be interpreted as being a form of ‘medical treatment’ so that it falls within the ambit of one of consent norms in the Children’ s Act, and therefore can be accessed independently by adolescents from the age of 12 onwards as a form of medical treatment [4, 20, 28]. The matter of parental consent has recently arisen in relation to vaccinations for COVID-19, causing similar debate [27].

Similarly to parental or caregiver buy-in, community acceptability of PrEP was a key enabler for implementation versus resistance to PrEP rollout in communities who were poorly informed around PrEP, or opposed to PrEP for moral or religious reasons. Opposition to PrEP from religious and faith leaders was cited as a challenge by some implementer respondents in our study. Socio-cultural norms and religious beliefs can play a part in either constraining or enabling individuals’ agency to engage in HIV prevention behaviours [29, 30]. Given the power that religious and faith leaders have to sway community opinions, it is critical to engage with community opinion leaders and faith leaders in efforts to promote acceptability for new HIV prevention tools such as PrEP, in order to create a conducive environment of AGYW’s successful uptake and adherence to PrEP. In our findings, implementers described various strategies that had been employed in attempt to improve acceptability and uptake of PrEP, including use of ‘PrEP ambassadors’. PrEP ‘ambassadors’ or ‘champions’, have been found to be a successful tool for disseminating information about PrEP to their peers and communities, and helping to dismantle PrEP stigma by deconstructing negative perceptions of PrEP and PrEP users, and thus fostering a more supportive social environment to enable PrEP use [31].

One key challenge cited by implementer respondents related to a lack of understanding and accurate knowledge about PrEP amongst parents and community members. Misinformation led to resistance towards PrEP provision to AGYW. Echoing findings in other studies conducted in the South African context [32], implementer respondents in our study described beliefs amongst community members and parents that taking PrEP would lead to HIV infection amongst AGYW, partly due to hearing about cases of seroconversion amongst PrEP users. Evident in our findings was also a narrative of resistance to PrEP based on mistrust fuelled by social media. Medical mistrust, encompassing a lack of trust in medical providers, the information they provide, and the medical system within which they function [33], is likely to be exacerbated within the context of COVID-19 vaccine hesitancy [34]. Studies show that high medical mistrust is associated with lower PrEP use willingness [33]. PrEP stigma and resistance are likely to be higher in contexts where community awareness about PrEP is low, which in turn acts as a considerable barrier to PrEP uptake and support for PrEP use amongst AGYW [12, 35].

PrEP stigma at multiple levels was evident in our findings, falling broadly into three categories: 1) public stigma due to perceptions of PrEP being treatment rather than prevention, and linked to this, 2) anticipated stigma amongst AGYW who feared being labelled as HIV positive for taking PrEP; 3) PrEP stigma related to assumptions of PrEP users as promiscuous / “high risk”. Relating to the first two, implementer respondents in our study described challenges related to PrEP being regarded as ART rather than prevention, and therefore assumptions made about PrEP users being HIV positive. This was particularly in cases where parents of AGYW were themselves HIV positive and on treatment; recognising the tablets, they would thus assume their daughter was also taking HIV treatment without their knowledge. Previous research shows that adherence to PrEP is negatively impacted by community-level stigma, which arises when PrEP users are assumed to be HIV positive due to taking a daily medication that is identical in appearance to commonly used oral HIV treatment tablets [13, 31, 35–37]. Several studies from across the sub-Saharan African context suggest that HIV related stigma arising from PrEP being conflated with ART acts as a barrier to PrEP uptake, continuation and acceptability, both as a form of anticipated or perceived stigma due to expectations of future judgement or discrimination by potential PrEP users’, and public stigma at the community level [23, 31, 37, 38].

Relating to the third stigma theme of PrEP being associated with high-risk behaviours and promiscuity, implementer respondents explained that community and parental resistance to PrEP was also due to beliefs that providing PrEP to AGYW condones their sexual behaviour, and encourages promiscuity. Parental concerns around the potential for increased sexual risk behaviours amongst adolescents and young people using PrEP have been discussed in the context of the United States, where parents’ fears related to the provision of PrEP being interpreted as an implicit approval for condomless sex have presented barriers to PrEP acceptability [39]. There may be some validity to these fears, given evidence suggesting that STI prevalence can increase with PrEP use, highlighting the importance of continued education around the importance of condoms in combination with PrEP [39]. The global framing of PrEP being targeted only to certain ‘high-risk’ population groups in clinical guidelines, and the persistent focus on risk in the framing of PrEP, enhances perceptions of PrEP being linked to risky sex, sexual irresponsibility and promiscuity, which in turn adds another aspect to PrEP related stigma [35, 37, 40]. Importantly, these aspects of PrEP stigma need to be understood within the context of socio-cultural norms around appropriate adolescent sexuality [35]. The South African government’s categorisation of AGYW as a high-risk population that should be prioritised for PrEP delivery, may have inadvertently fuelled healthcare providers’ already negative perceptions of adolescents’ sexuality. It had been argued that the continued implementation of risk-based assessments for PrEP eligibility serve to reinforce conceptual links between PrEP and riskiness/promiscuity, further compounding PrEP stigma and limiting access to it for individuals who are not deemed to be high risk [37].

Understanding the different types and causes of PrEP stigma, the various assumptions and negative perceptions of PrEP and PrEP users, is critical to address barriers to PrEP uptake, continuation and adherence [31]. Literature on PrEP stigma is limited, has largely centred on men who have sex with men (MSM), and has been focused on a Western context, but has clearly shown that PrEP stigma is associated with lower interest in and intention to use, discomfort discussing PrEP with service providers, and reduced PrEP uptake and adherence [36, 37]. There is a critical need to understand PrEP stigma in the sub-Sharan African context, and as it specifically relates to AGYW [35]. Our findings suggest that among South African AGYW, barriers towards PrEP acceptability are due to PrEP stigma manifesting at the individual level primarily as perceived or anticipated stigma, and external stigma at the public/community level.

### Limitations

Due to the scope and focus of this paper, we only include data on the perspectives and experiences of implementers of the intervention, and did not include the first-hand views of AGYW intervention beneficiaries, their parents, or other community stakeholders in this analysis, although these sample groups were included as respondents in the study. Additionally, since data collection was conducted at one time-point in the implementation of the intervention, as per the design of process evaluation studies, the data is limited to this particular time-point, and may not reflect adaptations made during the course of the programme. Lastly, is it possible that positive narratives about the intervention from implementer respondents may in part be due to social desirability reporting bias.

## Conclusions and Implications

Both societal and behavioural factors act as determinants of PrEP acceptability and use [38]. Our findings highlight the critical role of parental and community acceptability and support of PrEP as a key enabler in the successful PrEP demand creation, provision, uptake and adherence among South African AGYW. Adolescents are strongly influenced by the home and family environment, and therefore social support for PrEP use, and factors operating at the household, social and community levels, are critical in the successful enrolment, retention and adherence to PrEP amongst AGYW [12, 13, 32]. Given the evidence suggesting that peer influence and social support are potential facilitators to PrEP uptake and adherence amongst AGYW, programmes offering platforms for PrEP users to engage with and support each other could also be helpful [41].

A key aspect of increasing the availability and accessibility of PrEP to AGYW entails the expansion of education campaigns which can work towards raising community awareness around PrEP, and dispel confusion about PrEP as prevention rather than treatment. Messaging needs to ensure a positive framing of PrEP that not only works to create interest and improve demand creation amongst AGYW themselves, but also serves to build broader community awareness, trust and support for PrEP, in order to reduce stigma associated with using antiretrovirals for prevention and foster the necessary social support for PrEP use amongst AGYW [12, 14, 35]. Programmes comprising demand creation and community education campaigns, empowerment and social support interventions, and the promotion of adolescent-friendly healthcare services can help to facilitate disclosure of PrEP use, which is likely to positively impact uptake and adherence [35, 41]. The occurrence of diversion of PrEP medication for recreational or nonmedical use amongst AGYW in South Africa needs to be considered, highlighting the importance of pairing PrEP education campaigns with careful health communication messaging that supports proper use of PrEP, including the importance of adherence [42].

Community awareness campaigns to promote PrEP uptake and address resistance, misconceptions, and problematic social attitudes towards PrEP can help to dispel myths and problematic social perceptions of PrEP, and therefore address barriers to PrEP acceptability and uptake [9, 22, 35]. In order to address community resistance towards PrEP, which is often based on a lack of information, these campaigns need to be contextually appropriate and ‘culturally competent’ [43], informed by a comprehensive formative research approach, which enables an understanding of context specific barriers and facilitators to PrEP uptake and adherence in this population [44]. Contextually responsive and culturally appropriate interventions to shift social norms and views towards PrEP and adolescent sexuality more broadly, could be delivered through multi-level programmes offered in schools, healthcare settings, community centres and faith-based venues, and through the media by engaging trusted sources, opinion-leaders, influencers, peers and existing social networks [26, 32, 35, 43, 45]. Open dialogue and engagement with faith leaders to educate community gatekeepers about new HIV prevention options is critical, particularly in the sub-Saharan African context [30]. In addition, the engagement of men and boys is a critical factor in creating an enabling environment for PrEP use amongst AGYW, specifically efforts to address male perceptions that associate female PrEP use with promiscuity and mistrust [46].

Our findings highlight the critical importance of sufficient pre-counselling about how and when PrEP has sufficient protective coverage, alongside the provision of clear, accurate and simple information and infographics to AGYW, parents and communities, that illustrate concepts such as viral replication, mutation and drug resistance to make this point about the difference between antiretroviral therapy (ART), post-exposure prophylaxis (PEP) and PrEP. Shifting the narrative surrounding PrEP, to move away from the message that PrEP is for ‘high-risk’ groups, ‘key populations’, or promiscuous individuals, but rather for anyone in the general population who wants to empower themselves by protecting themselves and taking responsibility for their sexual health would help to de-stigmatise PrEP and thereby normalise its use [22, 32, 35, 36, 40]. Efforts to destigmatise PrEP and frame it as an empowering prevention tool that any responsible person can opt into at an appropriate time, could include rebranding with clear messaging and packaging to distinguish PrEP from ART, and offering PrEP alongside contraceptives, as part of an integrated broader package of SRH to AGYW, with a focus on promoting sexual health, pleasure, and intimacy [31, 35, 37, 41].

It is critical that implementation strategies to roll-out PrEP to AGYW be combined with messaging stressing the importance of condom use, to prevent other STIs and unintended pregnancies [39]. Additionally, PrEP programmes aimed at AGYW need to consider the lived realities of adolescents and young people in South Africa, and take into account user preferences for drug delivery and dosing, for example offering less user-dependent HIV prevention methods such as long-acting injectable PrEP rather than oral daily tablets, or intermittent versus daily use, in order to increase acceptability and reduce barriers to uptake, retention and adherence [1, 11, 48, 49]. In recognising the diversity of AGYW in South Africa, living across the socio-economic spectrum and facing different daily lived realities, adherence support approaches and incentives need to be tailored and responsive to this diversity [11, 13].

In addition to efforts to address issues relating to acceptability of PrEP, there is a need to address challenges with PrEP supply monitoring, forecasting, database management, distribution and delivery, as unreliable provision negatively impacts acceptability, uptake and retention. In order to mitigate supply interruptions, increase availability and access, enhance uptake and support PrEP use continuation amongst South African AGYW, innovative strategies are needed; a differentiated model could include flexible PrEP refill schedules, and various delivery options and distribution settings in addition to standard health facilities, such as mobile clinics and community-based venues, and even courier delivery services [10, 40, 47].

## Data Availability

The datasets used and/or analysed during the current study are available from the corresponding author on reasonable request.

## Footnotes

1 Peer group trainers are entry level staff, usually young people, who are responsible for delivering the core intervention. Care / outreach workers focus on support and advocacy on behalf of young people facing welfare, behavioural, developmental, social and protection issues, and are responsible for referrals and linkages. Coordinators at various levels are responsible for supporting and coordinating activities in the community, and establishing and maintaining partnerships with local organisations and stakeholders. Social auxiliary workers are responsible for individual and support group interventions, and managing peer group trainers. Social workers were involved in management and oversight, ensuring operational running of all behavioural programmes, and doing routine reporting. A range of managers were responsible for oversight, reporting and monitoring and evaluation, leading overall implementation, and managing stakeholder engagement. The programme will be supported by an administrator.
